# Inflammatory myofibroblastic tumor of the upper arm: A case report

**DOI:** 10.1097/MD.0000000000036558

**Published:** 2023-12-15

**Authors:** Caidi Yuan, Jie Fan, Lingjia Xu

**Affiliations:** a Department of Ultrasound, Shaoxing Second Hospital, Shaoxing, Zhejiang, China; b Department of Clinical Medicine, School of Medicine, Shaoxing University, Shaoxing, Zhejiang, China; c Department of Pathology, Huashan Hospital, Fudan University, Shanghai, China.

**Keywords:** brachial artery (BA), case reports, inflammatory pseudotumor, ultrasonography

## Abstract

**Rationale:**

Inflammatory myofibroblastic tumor (IMT) is an uncommon benign myofibroblastic tumor that usually occurs in the lung, mediastinum, abdomen and vulvovaginal region. IMT of the upper arm is exceedingly rare with unknown etiology. Pathology plays a major role in the diagnosis of IMT, and radiological characteristics of the condition are crucial for differential diagnosis.

**Patient concerns:**

A 62-year-old woman was admitted to our hospital for a complaint of a mass in her left upper limb with progressive numbness in the extremity. Ultrasound examination of the brachial artery (BA) revealed a hypoechoic mass with well-defined borders and a substantial blood flow, and the mass was also shown to be greatly enhanced on computed tomography (CT) and magnetic resonance imaging (MRI).

**Diagnosis:**

The subsequent histopathological and immunohistochemical studies led to the diagnosis of IMT.

**Intervention:**

The patient was referred for surgery. The soft tissue tumor resection, left median nerve release operation, brachial artery vascular grafting, and arterial anastomosis were performed.

**Outcome:**

Favorable outcome was observed. The patient recovered well from the procedure and did not experience any further complications or tumor recurrence.

**Lessons:**

In this report, we describe a case of IMT of the upper arm with BA involvement. The case expands the differential diagnosis of limb neoplasm and broadens the understanding of its ultrasonic and radiological imaging features. It also serves as a further example of an uncommon region distinct from conventional IMT. Further studies on the etiology and therapeutic strategies are needed.

## 1. Introduction

Inflammatory myofibroblastic tumor (IMT) is a relatively rare mesenchymal tumor exclusively affects the children and adolescents.^[1]^ It is a slow-growing, soft-tissue neoplasm characterized by intermediate biological behavior with a predilection for the lung, abdomen, retroperitoneum and pelvis,^[2]^ occasional cases have also been reported to arise in ciliary body, ventricle and ascending aorta.^[3–5]^ IMT treatment is nonspecific, challenging, and frequently necessitates surgical diagnosis.^[6]^ To our knowledge, there are some cases of IMTs detected in musculoskeletal system,^[7]^ timely diagnosis can help reduce complications and improve patients’ quality of life. However, IMT of the upper arm involving the brachial artery (BA) is clinically uncommon, we also found that the ultrasonography of IMT have rarely been reported.

Here, we report a case of IMT of the upper arm with BA involvement and provide a clear summary of the ultrasonic imaging results with the goal of increasing the understanding and experience of ultrasonic and radiological knowledge in the differential diagnosis of this tumor.

## 2. Case report

A 62-year-old woman with no underlying medical conditions, family history of genetic diseases or traumatic history, was admitted to our hospital for a complaint of a mass of her left upper limb with progressive numbness in the extremity for 4 years. The mass was superficial and grew slowly. Physical examination on admission found a tough and irregular palpable mass of the left upper limb, about 1.5 cm × 1.5 cm, with unclear boundaries and poor mobility. The left radial artery pulsation disappeared when pressing the mass. Sensation deficits of the left 3 lateral digits including their nail beds were detected but there was no obvious abnormality in the movement of the left upper limb and no more than 20 mmHg difference between the right and left upper arm blood pressure. The indicators of her laboratory inspections such as blood routine test, coagulation function, liver and kidney function, viral serology and tumor-related markers were all within the normal range.

**Figure 1.**
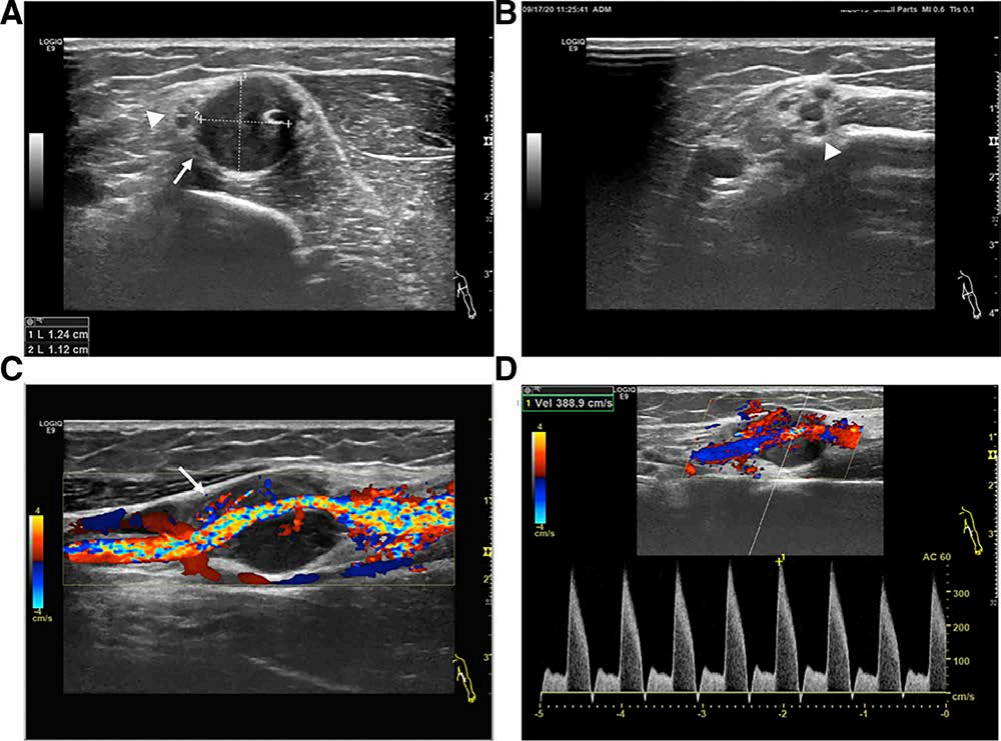
Ultrasonography of the left brachial artery (BA). (A, B) A hypoechoic mass in the middle layer of the left BA (12 × 11mm), with regular morphology and clear boundaries (white arrow). The peripheral median nerve is compressed with edema (white arrowheads). (C) Longitudinal section examination reveals a hypoechoic mass between the outer wall and the lumen of the vessel with abundant blood flow running through, and the lumen is narrowed and distorted (white arrow). (D) Doppler ultrasound detects severe stenosis of the BA (the peak speed is 389cm/s).

**Figure 2.**
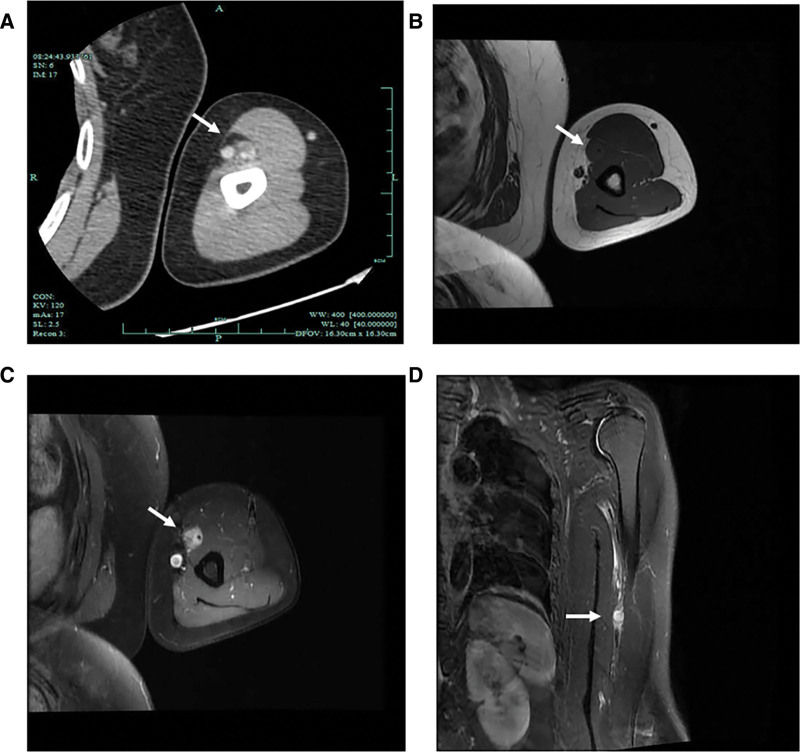
Computed tomography (CT) and magnetic resonance imaging (MRI) of the tumor. (A) Contrast-enhanced CT of the left upper limb shows nodular soft tissue density shadow inside the left upper arm with significantly enhancement, and the demarcation between the lesion and surrounding soft tissue is clear, closely adjacent to the BA (white arrow). (B–D) MRI demonstrates the lesion is moderate intensity but significantly enhanced on T1-weighted imaging and has high intensity in T2 fat suppressed sequence (white arrows).

Doppler ultrasound examination found a hypoechoic mass (12 mm × 11 mm) with clear boundaries adjacent to the wall of the BA (Fig. 1A). The peripheral median nerve was compressed and thickened with edema (Fig. 1A and B). Longitudinal section examination showed blood flow of the BA run through the hypoechoic mass which located between the outer wall and the lumen of the blood vessel with no obvious capsule, and the brachial lumen was compressed and distorted (Fig. 1C). Color Doppler ultrasound examination detected severe narrowing of the BA (Fig. 1D). Computed tomography (CT) demonstrated the lesion was circular with marked enhancement, the demarcation between the lesion and the surrounding soft tissue was clear, and it was closely adjacent to the BA (Fig. 2A), magnetic resonance imaging (MRI) found a kind of circular abnormal signal shadow in the biceps space in the middle of the left upper limb, showing moderate intensity but significantly enhanced on T1-weighted imaging and high intensity on T2 fat suppressed sequence, and the lesion was closely related to the adjacent blood vessels. The left humerus was morphologically normal, and there were no obvious abnormal signal foci in the bone. (Fig. 2B–D). These findings led to the possible diagnosis of angioma in BA.

**Figure 3.**
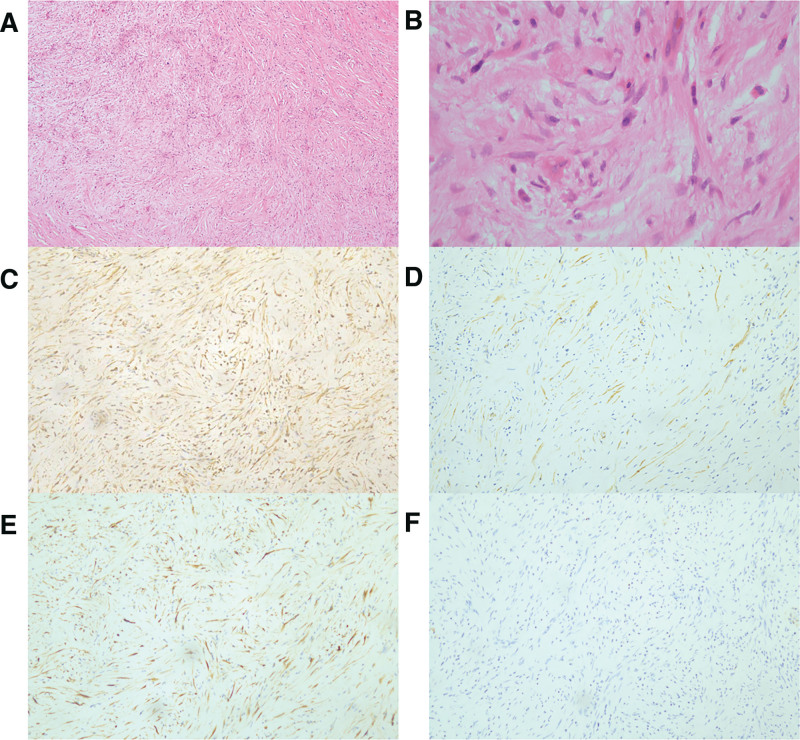
Pathological examination and immunohistochemical staining. (A) Short fascicles of spindled myofibroblastic cells without overt atypia, hematoxylin-eosin staining (HE), magnification × 40; (B) HE, magnification × 100; (C) Vimentin positivity, magnification × 100; (D) SMA positivity, magnification × 100; (E) ALK negativity, magnification × 100; (F) Desmin negativity, magnification × 100.

The patient declined the suggested biopsy and was referred for surgery. The soft tissue tumor resection, left median nerve release operation, brachial artery vascular grafting, and arterial anastomosis were performed with no surgical complications. The specimens were found to be firm, sharply demarcated, and grayish white to yellow upon pathological inspection. Under the microscope, there were several myofibroblastic-like spindle cells mixed in with different inflammatory cells that had infiltrated. There was no evidence of spindle cell dysplasia, and the mitotic activity was minimal (Supplementary Figure 1, http://links.lww.com/MD/L32; Fig. 3A and B). Immunohistochemical analysis (Fig. 3C–F) revealed positive staining results for vimentin, smooth muscle actin (SMA), CD34, and elastic fiber, as well as negative staining results for CK, Ki-67 (3%), desmin and β-cat. Interphase fluorescence in situ hybridization (FISH) test was performed for anaplastic lymphoma kinase (ALK) rearrangement and the result turned out to be negative. The final diagnosis based on histological and immunohistochemical assessments was IMT.

**Figure 4:**
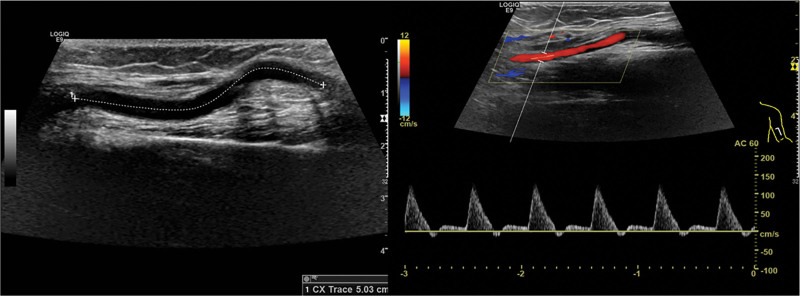
. Postoperative follow-up ultrasonography. The self-grafted blood artery was curved and patency.

Considering the low-grade IMT histological diagnosis, no further treatment was administered. The patient had no more complications and recovered well from the surgery. A doppler ultrasonography performed a year later revealed that the self-grafted blood artery was curved and patency with no tumor recurrence. (Fig. 4 and Supplementary Figure 2, http://links.lww.com/MD/L33 which indicates the timeline).

## 3. Discussion

IMT is a rare benign myofibroblastic tumor that can occur in any part of the body, most commonly in the lung, abdomen, mesentery and retroperitoneum,^[8]^ According to a recent review by Siemion et al,^[9]^ 75% of these cases occur in the abdominal cavity, and peripheral artery or nerve involvement are exceedingly rare. In the present case, the distinct feature of this instance is that it immediately adjacent to wall of the BA and involves the median nerve. The pathogenesis of this condition may be attributed to various factors such as trauma, surgical procedures, abnormal repair processes, autoimmunity, and infection. However, the precise cause and etiology of the majority of cases remain unidentified. Additionally, there is no documented history of potential illness in this particular case. The infiltration of inflammatory cells and fibroblast hyperplasia of the vascular wall, along with an uncommon mitotic phase that is specific to high-grade malignancies, are the pathogenic features. It has been reported that the upstream frameshift 1 mutation downregulates nonsense-mediated RNA decay within the molecular pathways, this suppressed expression of NF-κB then plays a role in the immune system ability to infiltrate IMT signs.^[10]^ Lee et al^[7]^reported a case of ALK-negative IMT in the triceps brachii which grew rapidly, resulting in ulnar nerve compression symptoms in this patient, MRI features were similar to those in our case, the tumor was surgically resected and the patient condition improved with no recurrence after 12-month follow-up. Another case involved an IMT in the forearm that resulted in posterior interosseous nerve palsy.^[11]^ The patient was initially misdiagnosed as lateral tendinopathy, but an ultrasonic and MRI examination eventually revealed the presence of a mass near the radial nerve, extending from the proximal elbow to the supinator muscle outlet; following 3 months of ineffective conservative treatment, the patient symptoms were significantly alleviated after surgery. Additionally, in certain cases affecting the musculoskeletal system, a radiographic examination must be done promptly to facilitate a differential diagnosis. Even though IMT appears as a benign tumor, surgical therapy can improve the prognosis since it frequently produces compressive impacts.

IMT has no specific symptoms and signs, so preoperative diagnosis is difficult. Clinical imaging characteristics of IMT are rarely been reported but essential for differential diagnosis. Based on the pathological histology of IMT, it is frequently diagnosed with solitary hypoechoic or moderate-echoic masses that have regular form, defined borders, unequal internal echo, and no appreciably attenuated or amplified posterior echo on ultrasonography. Abundant blood flow signals related to inflammation can be seen inside. The tumor can compress or strain its peripheral blood vessels and nerves, although it tends to grow slowly without distant metastasis or recurrence. On CT imaging, IMT manifests as well-defined homogeneous intermediate intensity and moderate to strong enhancement. On MRI, homogenous hypo-intensity would be shown on T1, and enhancement would be found following the administration of contrast, while high-intensity observed on T2 fat suppressed sequence. IMT is rarely diagnosed based on ultrasonic and radiologic imaging but they have certain significance for differential diagnosis.

IMT has similar histologic morphology and immunologic phenotype to many soft tissue neoplasms, but the biological behavior and the clinical management is extremely different, so differential diagnosis is especially significant. Angiofibromas are commonly found in the nasal cavity, orbit and eyelids.^[12]^ Microscopically, it is shown as short spindle cells with unequal distributed and diffused thin-walled lobulated blood vessels in a mucous or glioidal background, on immunophenotyping, it expresses vimentin, CD31, CD34, in addition to myogenic antibodies.^[13]^ Schwannoma is a benign tumor originating from Schwan cells that encases the peripheral nerves with an intact envelope. It may occasionally arise in peripheral nerves of the limbs. Schwannoma is more prone to focal bleeding, necrosis, and cystic degeneration, and the nearby nerves at both ends show a specific manifestation of “rat tail sign” on ultrasonography for identification.^[14]^ Leiomyomas are more common in middle-aged and elderly patients, arise in the pelvis, retroperitoneal and abdominal cavity.^[15]^ The morphology under the microscope is similar to that of IMT, however immunohistochemical expression of myogenic antibodies such as SMA is positive, and negative ALK can be distinguished from IMT.

The treatment of IMT should be resected surgically and rapid frozen section pathological examination should be performed during surgery. The surgical method and resection scope should be determined according to the intraoperative exploration and pathological results. On the basis of excision of the diseased tissue, normal tissue must be preserved as much as possible. It is thought that the effects of conventional postoperative chemotherapy and/or radiotherapy are indefinite.^[16]^ Radical excisional surgery continues to be regarded as the optimal therapeutic approach, which has an overall favorable prognosis with a 5-year event-free survival and overall survival of 82.9% and 98.1%, respectively.^[17]^ Overall, the metastasis rate is <5% and recurrence rates range from 2% to 60%.^[18]^ No metastasis recurrence has been detected so far in this patient at postoperative follow-up.

## 4. Conclusion

Here, we describe a rare case of IMT of the upper arm with BA involvement and summarized its imaging characteristics. The ultrasonic imaging signs play a key role in the differential diagnosis of IMT. The tumor tends to grow slowly without distant metastasis or recurrence but can develop a squeeze or compress effect, surgical removal of the tumor while sparing adjacent nerves as much as possible is the preferred treatment and the prognosis is favorable.

## Author contributions

**Conceptualization:** Caidi Yuan.

**Data curation:** Lingjia Xu.

**Investigation:** Caidi Yuan, Jie Fan.

**Supervision:** Lingjia Xu.

**Validation:** Lingjia Xu.

**Writing – original draft:** Caidi Yuan.

**Writing – review & editing:** Jie Fan, Lingjia Xu.

## Supplementary Material




