# Supplementary figures and images for: Inflammatory myofibroblastic tumor of the upper arm: A case report
Source: Medicine (Baltimore). 2023 Dec 15;102(50):e36558. doi: 10.1097/MD.0000000000036558 (PMC10727551; doi:10.1097/MD.0000000000036558)

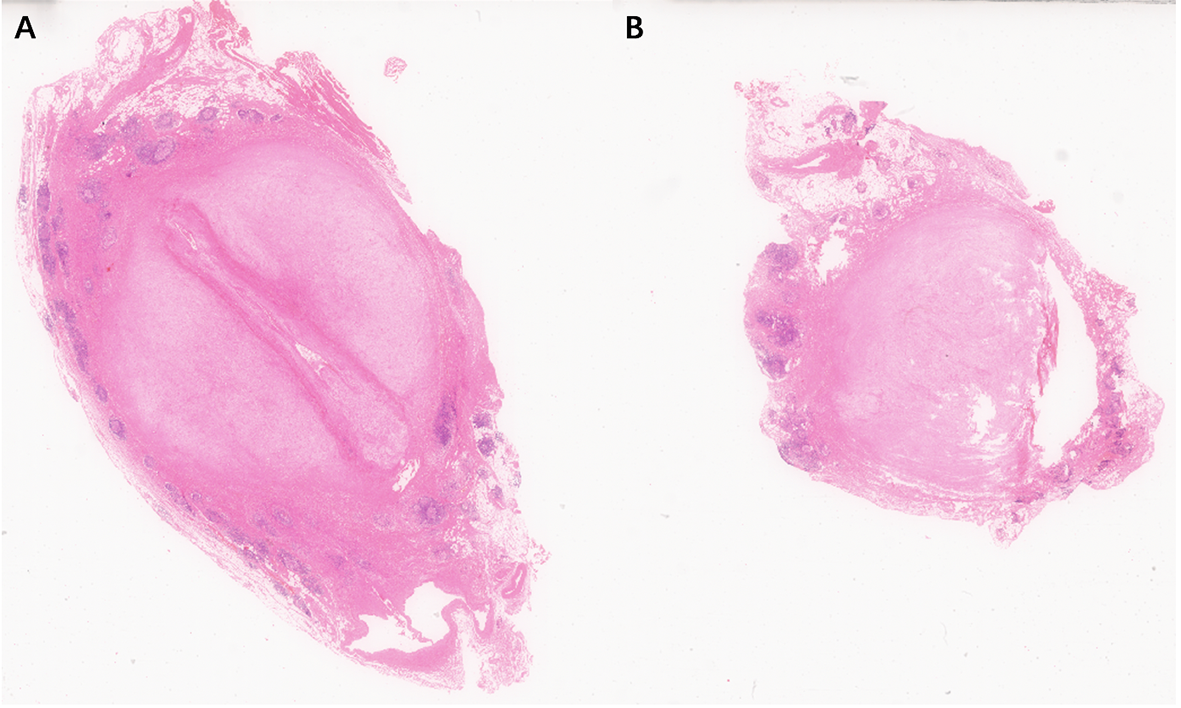

Supplement: Supplementary file 1 [file medi-102-e36558-s001.tif]

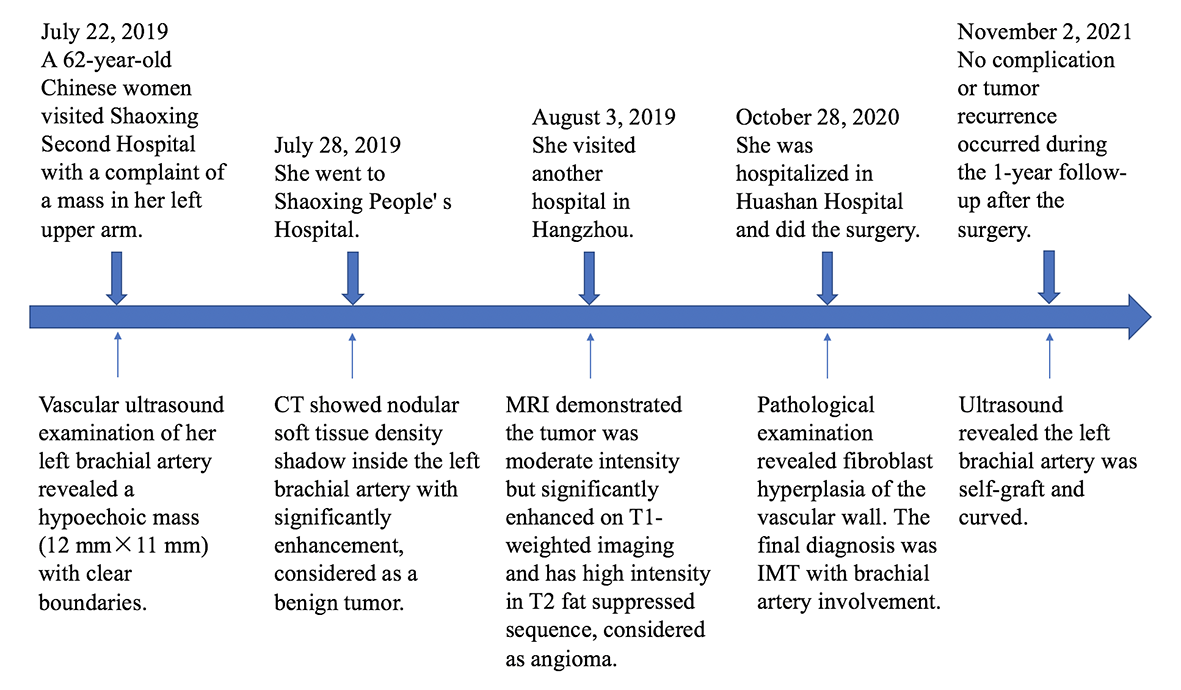

Supplement: Supplementary file 2 [file medi-102-e36558-s002.tif]
